# Direct Age Determination of a Subtropical Freshwater Crayfish (Redclaw, *Cherax quadricarinatus*) Using Ossicular Growth Marks

**DOI:** 10.1371/journal.pone.0134966

**Published:** 2015-08-26

**Authors:** Jesse C. Leland, Daniel J. Bucher, Jason Coughran

**Affiliations:** 1 Marine Ecology Research Centre, School of Environment, Science and Engineering, Southern Cross University, Lismore, New South Wales, 2480, Australia; 2 Faculty of Mathematics, Physical Sciences and Life Sciences, Sheridan College, Perth, 6000, Western Australia, Australia; Department of Agriculture, AUSTRALIA

## Abstract

Recent studies have reported that crustacean age determination is possible. We applied a direct ageing method (i.e. transverse cross sectioning of gastric ossicles) to a subtropical freshwater crayfish (*Cherax quadricarinatus*) sourced from an aquaculture population. Growth mark periodicity and the potential for chronological depositions were investigated by staining *C*. *quadricarinatus* with calcein and examining their ossicles a year later. Pterocardiac ossicles were superior to other ageing structures (i.e. other ossicles and eyestalks) and produced repeatable between-reader counts (87% were corroborated and 13% varied by ±1). *C*. *quadricarinatus* size-at-age data (for an aquaculture population) was described by a von Bertalanffy growth equation (*L*_*∞*_ = 32 mm occipital carapace length; *K* = 0.64; *t*_0_ = –0.18; R^2^ = 0.81). Ossicular growth marks did not correspond to moult history. The calcein stain was retained over an annual cycle comprising multiple moults, demonstrating that pterocardiac ossicles retain chronological information. The maximum age (3+) corroborated other indirectly-obtained longevity estimates for *C*. *quadricarinatus*. Multiple lines of evidence indicate that the growth marks in *C*. *quadricarinatus* ossicles are probably deposited annually during winter. The ability to extract age information from subtropical decapods provides substantial opportunities for advancing fisheries and conservation research globally, but further research is needed to provide a definitive validation and elucidate the mechanism governing the accrual of ossicular growth marks.

## Introduction

Age information is important for calculating growth, mortality and productivity [1] and is therefore important for both fisheries and conservation management [2–3]. Because of this, substantial research efforts have focused on developing direct age determination methodologies for aquatic species. Consequently, analyses of sequentially-deposited growth marks in calcified structures including fish otoliths and mollusc shells [4–5] have become routine. However, the development of analogous techniques for ageing crustaceans has lagged behind. Until recently, it was presumed that moulting precluded the possibility of growth record retention [6] and this untested assumption stymied the development of direct ageing techniques. As a result, crustacean ageing studies use comparatively inferior indirect methods (e.g. size-frequency, tag-and-recapture and lipofuscin accumulation [6]) that are useful, but cannot produce repeatable and independently verified (i.e. ‘validated’ [1]) age estimates for individuals [7–8].

Leland et al. [7] identified sequential growth marks in sectioned gastric ossicles from a range of crayfish (i.e. redclaw, *Cherax quadricarinatus* and powerful crayfish, *Euastacus valentulus*), crab (i.e. mud crab, *Scylla serrata* and spanner crab, *Ranina ranina*) and lobster (i.e. Moreton Bay bug, *Thenus orientalis*) species. Subsequently, other studies have provided strong evidence that the growth marks in temperate crustaceans probably correspond to annual growth cycles [8–10], but detailed studies from subtropical regions are lacking. Compared with temperate fish, seasonally-driven growth variations in subtropical taxa can be relatively less marked, sometimes resulting in reduced otolith readability [11] or bi-annual periodicity [12]. Therefore, periodicity validation is required on a region-specific and species-by-species basis [1]. One commonly used validation method involves applying chemical stains that produce a known-date artificial mark, with subsequent growth being assessed over an annual cycle [1, 4]. Calcein is a fluorescent dye that is effective for marking numerous ageing structures [13–14] and was used to tag American lobster (*Homarus americanus*) for up to six months [8].

Crustacean ageing is understudied with < 2% of the world’s decapods having reliable longevity information available [6]. The infraorder Astacidea includes some particularly long-lived freshwater crayfish [6, 15], including some that are commercially and/or recreationally exploited (e.g. in Australia and Europe [16–17]). In addition, many freshwater astacids are threatened by habitat degradation, invasive species and climate change [18]. Despite the apparent longevity among some freshwater astacids [6] and their fisheries and conservation importance, validated ageing studies do not exist.

Australia’s freshwater crayfish fauna include some common and widely-distributed taxa (e.g. *C*. *quadricarinatus* [19]) and others that are comparatively rare with extremely restricted ranges [20]. Similar contrasts are also apparent in age and longevity characteristics [6]. For example, indirect ageing of *Euastacus* report longevities of >25 years for *E*. *armatus* [16] and *E*. *sulcatus* [15], while other species like *C*. *quadricarinatus* are relatively fast growing [19] and short-lived (i.e. 3–5 years [21–22]). *Cherax quadricarinatus* is native to the Australian and New Guinean tropics [19], but has been translocated to subtropical regions of eastern Australia [23], the Americas, Europe, Asia and the Middle East [24].

The relatively short life span, rapid growth and wide geographic distribution of redclaw crayfish make it an ideal model species for age determination studies. We hypothesise that ossicular marks comprise a record of seasonal growth rate changes that are therefore suitable for direct age determination. This hypothesis was investigated by applying a flurochrome stain (calcein) to *C*. *quadricarinatus*. After staining *C*. *quadricarinatus* were reared for one year, before determination of calcein retention and subsequent ossicular deposition was made. If the growth marks are annual then only a single translucent-opaque cycle (i.e. summer-winter) should be deposited beyond the calcein and the maximum putative age should corroborate indirect longevity estimates.

## Materials and Methods

### Study specimens and sample preparation

Live *C*. *quadricarinatus* were sourced from a commercial aquaculture facility (Cherax Park Aquaculture Farm in Theebine, Queensland)–where they are reared in outdoor ponds–before being transported to Southern Cross University (Lismore, New South Wales) on April 26 2013. *Cherax quadricarinatus* for age estimation were sacrificed immediately, while others were retained live for the calcein staining and grow-out (see below). All individuals were sexed and measured for occipital carapace length (OCL–mm [23]). During dissection *C*. *quadricarinatus* were moult-staged and placed into pre-moult and intermoult categories, based on gastrolith presence or absence respectively. An animal ethics permit was not required for this study, because invertebrate crayfish are exempt under the relevant national code (Australian code for the care and use of animals for scientific purposes 8^th^ Edition, 2013) and NSW state legislation (Animal Research Act 1985 No. 123). All crayfish were sacrificed by placement into an ice slurry or a laboratory freezer.

**Fig 1:**
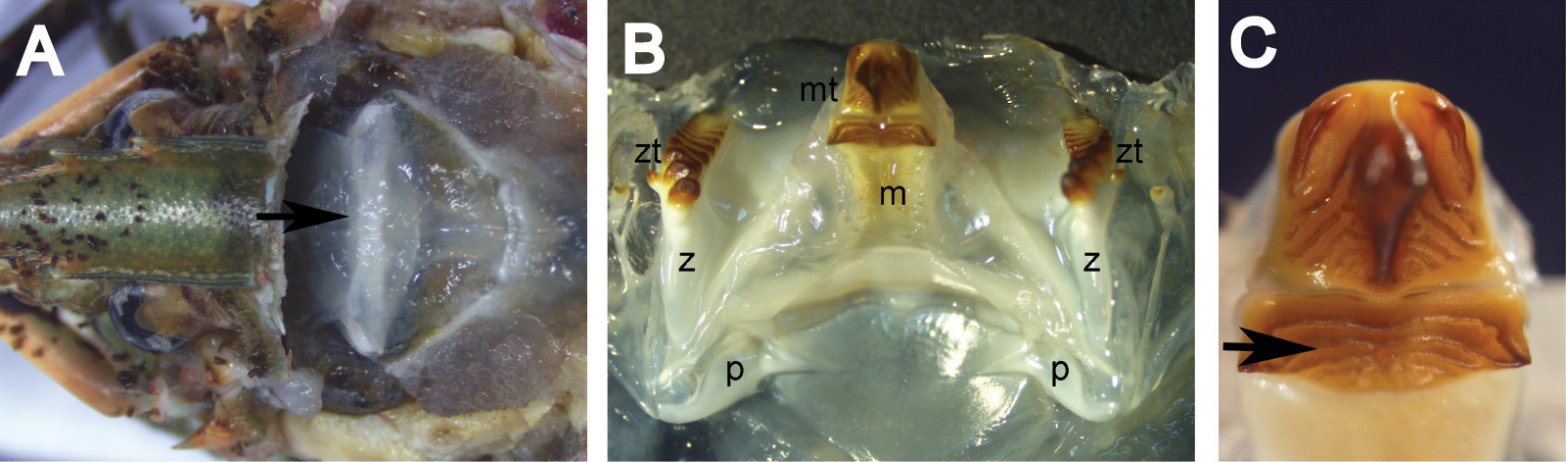
The gastric mill and its components. A: *Cherax quadricarinatus* specimen with the carapace partly removed to expose the cardiac stomach containing the gastric mill (indicated by the black arrow). B: *Cherax quadricarinatus* gastric mill showing the structural arrangement of the single mesocardiac (m) ossicle and paired zygocardiac (z) and pterocardiac (p) ossicles. The mesocardiac and zygocardiac ossicles support distally-positioned tooth plates (mt = mesocardiac tooth, zt = zygocardiac teeth) that are used to grind food items. The tooth plates are directed posteriorly within the cardiac stomach. C: Close up of the mesocardiac tooth plate showing the region containing urocardiac ridges (indicated by the black arrow).

**Fig 2:**
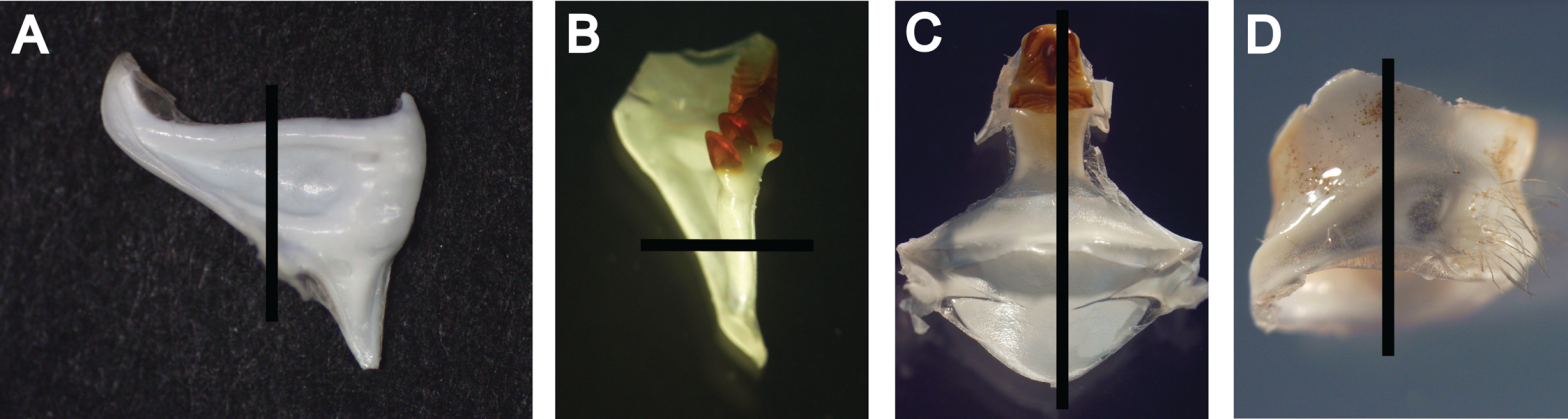
The disarticulated ossicles and eyestalk of *C*. *quadricarinatus* showing the sectioning plane (indicated by the black line) and positioning for each structure. A: Pterocardiac ossicles were sectioned transversely at the approximate midpoint of the lateral edge (top of photo). B: Zygocardiac ossicles were sectioned transversely at the approximate midpoint between the first tooth and the attachment point. C: Mesocardiac ossicles were sectioned longitudinally, with the placement being intentionally and slightly off centre (by ~200 μm). D: Eyestalks were sectioned longitudinally.

Gastric mills were extracted whole (Fig 1A and 1B) from only intact (i.e. without any missing appendages) *C*. *quadricarinatus* and their mesocardiac, zygocardiac and pterocardiac ossicles were disarticulated. Eyestalks were excised whole and all soft tissues were removed. Ossicles and eyestalks were rinsed with distilled water and stored in a 60:30:10 mix of ethanol, glycerol and water. Before embedding, the number of zygocardiac teeth and urocardiac ridges (as described by [25]) (Fig 1C) was counted and both structures were submerged in 100% ethanol (~5 s) and air dried (~1 h). Ossicles and eyestalks were embedded in polyester resin and sectioned (200 μm thickness) with a Buehler Isomet saw. Centrally-positioned transverse cross sections were taken from pterocardiac and zygocardiac ossicles (Fig 2A and 2B), while mesocardiac ossicles and eyestalks were cut longitudinally (Fig 2C and 2D) (following Leland et al. [7] for zygo- and mesocardiac ossicles and Kilada et al. [8] for eyestalks). All sections were stored dry.

### Age estimation, repeatability and growth modelling

A preliminary assessment showed that all three ossicles contained alternating translucent and opaque zones (i.e. termed ‘growth marks’), but these were not found in eyestalks. Pterocardiac ossicles were chosen for age estimation, because of their superior readability. The exo- and endocuticle layers (as termed by [8]) were visually distinct and easily identified under light microscopy. For all subsequent age estimates, counts originated from the interface between these two layers (termed the ‘cuticular boundary’). Ossicular sections were wetted with distilled water and placed on glass slides for imaging under an Olympus CX40 compound microscope (at 20–40×) fit with a DP12 digital camera. Each section was photographed using analySIS Five software (Life Science), before measurements of total ossicle width were taken using the arbitrary distance function (nearest 0.1 μm). The number of lamellae present within the exocuticle was counted from digital images.

Each *C*. *quadricarinatus* was assigned a putative age category based on the number of primary growth marks within the endocuticle (i.e. ‘0+’, ‘1+’, ‘2+’ or ‘3+’). Ossicle readability was categorised as either ‘good’ (i.e. having well-defined and easily read primary marks), ‘intermediate’ (i.e. having readable but relatively poorly-defined primary marks) or ‘poor’ (i.e. the section was unreadable) and compared with moult stage to assess any potential inter-relationship. Putative ages were adjusted (+0.5 years) to account for the time elapsed between hatching (late spring–early summer, estimated as November 1) and collection. All sections were re-read (as above) by a second observer (without knowledge of the previous count) to determine count repeatability (expressed as percentage agreement). All counts were done without knowledge of the individual’s size.

Linear regressions were fitted to morphometric data using the Statistical Package for Social Sciences (SPSS) version 22 (IBM Corporation). Occipital carapace length-at-age data were fitted with a von Bertalanffy growth model using the SPSS non-linear regression function: OCL_t_ = *L*_*∞*_ [1 –e −^K (t–t^_0_^)^], where *L*_*∞*_ is the asymptotic OCL at infinite age, *K* is the Brody growth constant affecting curvature and *t*_0_ is the hypothetical age at zero OCL. The iteration starting values for *L*_*∞*_ and *K* (i.e. 31 mm OCL and 0.18) were nominated and adjusting these did not affect the final *L*_*∞*_ and *K* estimates.

### Calcein staining, grow-out and detection

Freshwater and calcein were combined (at 500 mg l^-1^) in an aerated aquarium. The pH was adjusted upwards (to 7.5) using 1M NaOH. Fifty-five *C*. *quadricarinatus* were stained by immersion (for 72 h) during late April–early May 2013. All *C*. *quadricarinatus* were housed individually and reared for up to one year (termed ‘grow-out period’) under ambient temperatures (with light fixed at 12:12 h). During the grow-out period *C*. *quadricarinatus* were fed commercial aquaculture pellets (ad libitum) periodically supplemented with algae and organic debris (i.e. leaves and sticks). The foregut exuviae of stained individuals that moulted were examined for gastric ossicles.

A randomly-chosen subset of the stained individuals was sacrificed after 10 months (*n* = 2) and one year (*n* = 7). Stained ossicles were prepared, embedded and sectioned (as above) for calcein detection. Stained-ossicle sections were viewed (at 10–40×) under a Nikon A1R confocal microscope (Laser 4.2, HV 110–118). Confocal microscopy images were taken using two different emission wavelengths (420 and 590 nm for green and red, respectively) and NIS Elements software (AR410.00).

## Results

### Age estimation, repeatability and growth modelling

**Fig 3:**
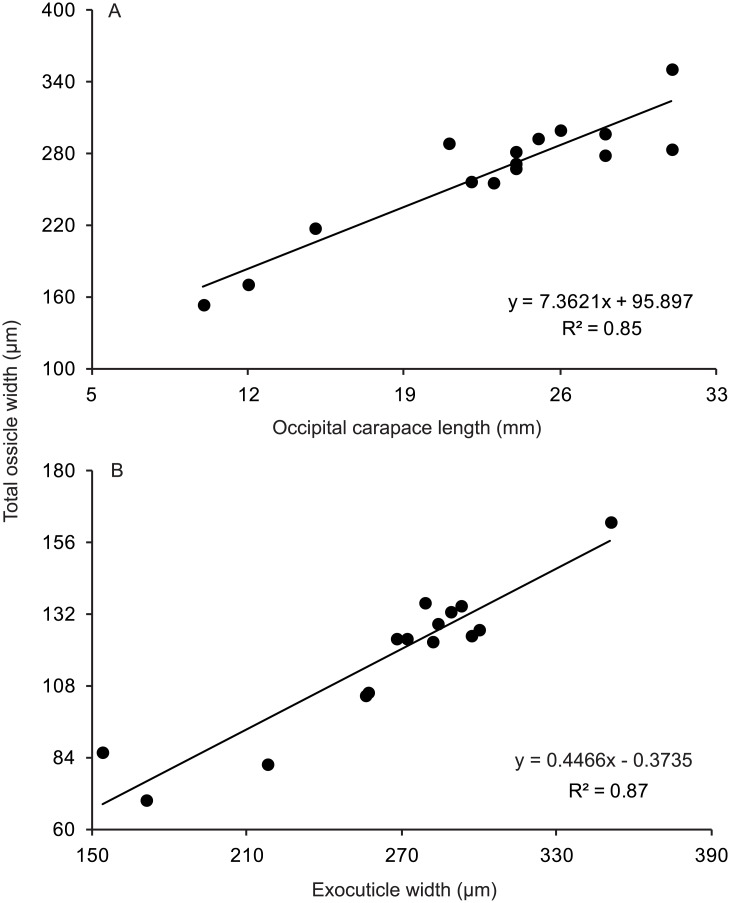
Linear relationships established for *C*. *quadricarinatus*. A: Occipital carapace length and total ossicle width (*n* = 15). B: Exocuticle width and total ossicle width (*n* = 15).

The exocuticular layer contained regular lamellae (13–18), the number of which was only weakly correlated with OCL (y = 0.054x + 13.703; R^2^ = 0.02; *n* = 12). Occipital carapace length was very strongly correlated with total ossicle width (Fig 3A). Exocuticle width was very strongly correlated with that of the ossicle (Fig 3B).

**Fig 4:**
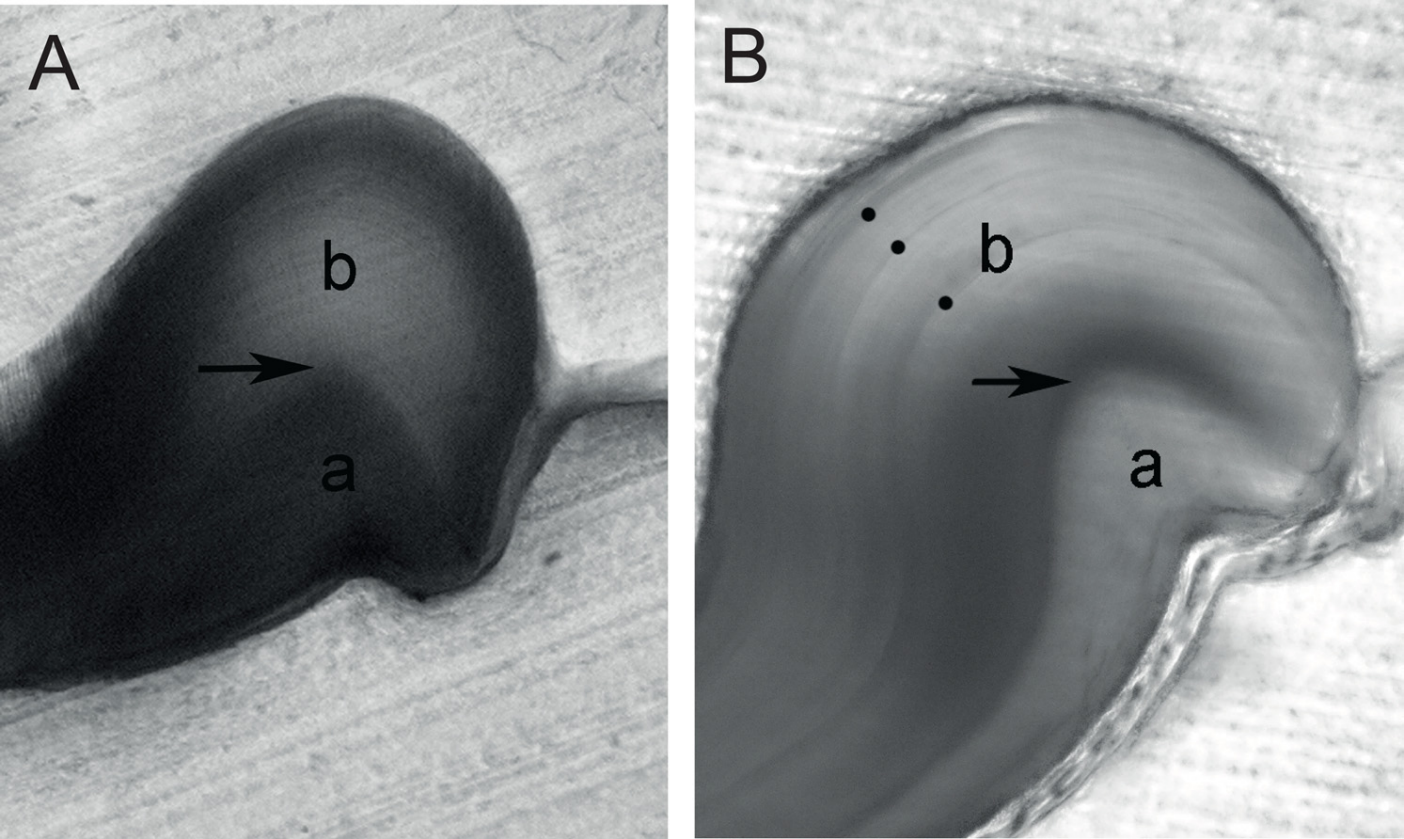
Pterocardiac ossicles used to assign *Cherax quadricarinatus* putative ages. The exocuticular (a) and endocuticular (b) layers are divided by the cuticular boundary (indicated by the black arrow). Growth marks only occur within the endocuticle. A: Juvenile aged at 0+ (6 months), without a growth mark beyond the cuticular boundary. B: Adult crayfish aged at 3+ (3.5 years), showing declining spacing between endocuticular growth marks (indicated by black dots). Note the presence of additional material (> 50% of the previous cycle) beyond the outermost growth mark.

**Fig 5:**
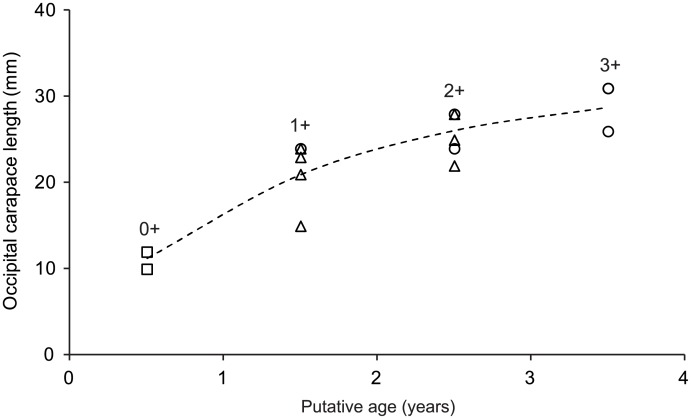
*Cherax quadricarinatus* occipital carapace length for 0–3+ putative age classes fitted with a von Bertalanffy growth curve. Putative ages were estimated based on the number of primary growth marks within the endocuticle. The von Bertalanffy growth parameters for the aquaculture population sampled were: *L*_*∞*_ = 32 mm OCL; *K* = 0.64; *t*_0_ = –0.18; R^2^ = 0.81; *n* = 15). Square = juvenile, circle = male, triangle = female.

The two smallest individuals (10 and 12 mm OCL juveniles) had easily identified cuticular boundaries, but no subsequent growth mark within the endocuticle and were aged at 0+ (Fig 4A). All remaining *C*. *quadricarinatus* (15–31 mm OCL adults) had between 1 and 3 concentrically-arranged growth marks occurring within the endocuticle (Fig 4B). For all sections, the distance between adjacent marks declined with each successive mark and ossicular material (> 50% of the previous cycle) was present beyond the outer-most mark (Fig 4B). The von Bertalanffy equation fitted the size-at-age data better (*L*_*∞*_ = 32 mm OCL; *K* = 0.64; *t*_0_ = –0.18; R^2^ = 0.81; *n* = 15; Fig 5) than a linear model (y = 5.485x + 11.414; R^2^ = 0.74; *n* = 15). There was no obvious difference between male and female size-at-age, but the three oldest individuals (3+) were all male (Fig 5).

Independent readings (*n* = 15) demonstrated that counts were repeatable, with most being corroborated (87%) by the second observer or ±1 (13%). There was no apparent relationship between section readability and count repeatability or moult stage. Two sections were categorised as poor and discarded.

### Morphological observations

**Fig 6:**
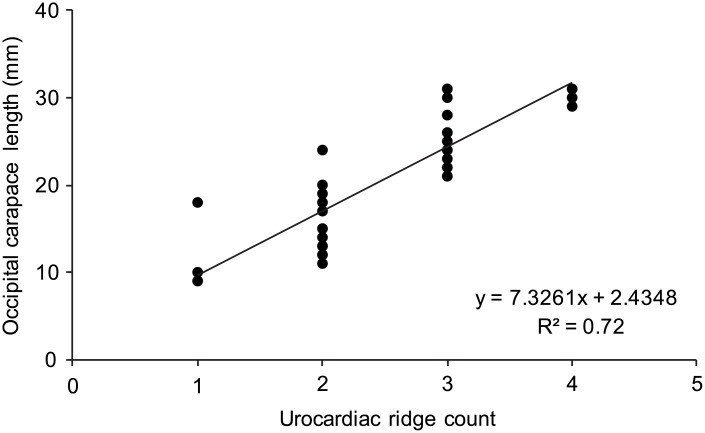
Relationship between the number of urocardiac ridges and occipital carapace length for *Cherax quadricarinatus*. The number of urocardiac ridges increases with increasing crayfish size (*n* = 34).

Irrespective of size, the number of zygocardiac teeth (8–10) was similar and weakly correlated with OCL (y = –1.995x + 37.90; R^2^ = 0.04; *n* = 37). The number of urocardiac ridges on the mesocardiac tooth plate was strongly and positively correlated with OCL, with small *C*. *quadricarinatus* having a single ridge and larger crayfish having up to four (Fig 6). *C*. *quadricarinatus* foregut exuviae (*n* = 50) always contained only the shed meso- and zygocardiac tooth plates (i.e. without any trace of the corresponding ossicles).

### Calcein staining, grow-out and retention

One *C*. *quadricarinatus* (< 2%) died during the staining procedure, but seven other fatalities (< 13%) occurred during the grow-out. During that time, 34 *C*. *quadricarinatus* moulted (from 1–6 times). Most moults (*n* = 23) occurred during summer (December–February), but 10 moults occurred in winter (June–August). The calcein stain was retained in the meso- and zygocardiac ossicles of the two *C*. *quadricarinatus* (18 and 24 mm OCL) sacrificed after 10 months (both individuals had moulted once).

**Fig 7:**
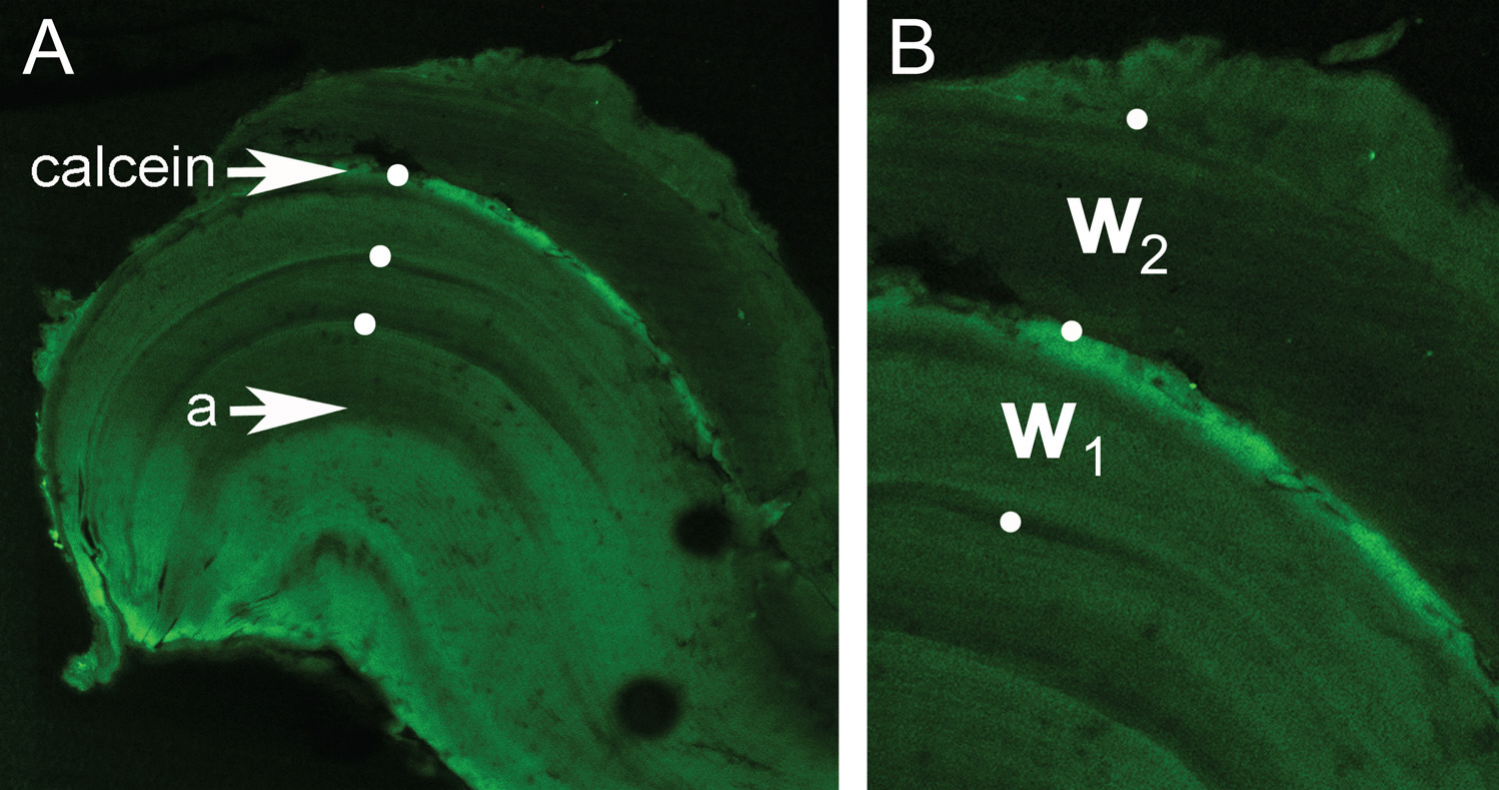
Confocal microscopy image showing a *Cherax quadricarinatus* ossicle from a 3+ crayfish that was stained with calcein and reared for one year. A: The cuticular boundary (a) and calcein stain incorporated just before and adjacent to the third growth mark (indicated by white dots). B: Inset showing the relative width of the growth cycle incorporating the calcein mark (w_1_) and the subsequent annual cycle (w_2_).

Seven other individuals were sacrificed after one year. Of these, all had moulted and three *C*. *quadricarinatus* (that moulted 1, 3 or 4 times) had an easily identifiable calcein mark within the endocuticle, but the amount of subsequent growth varied substantially. One large adult *C*. *quadricarinatus* showed approximately a year’s proportional growth i.e., the width of the material deposited during the 12 months laboratory grow-out was 110% of the previous cycle’s width (Fig 7A and 7B), which was produced in outdoor ponds. In this individual, a discrete calcein mark was positioned within the endocuticle and just before the third growth mark (Fig 7A and 7B). For the remaining four individuals, the position of the calcein was either along the outer edge or a discrete mark was not clearly distinguishable.

## Discussion

Recent methodological advances [7–8] have allowed for hitherto impossible direct age determination and periodicity assessment. This study provides the first assessment of stain retention and subsequent ossicular growth over an annual cycle for any crustacean. We give the first direct evidence (but see [8–10]) for annual periodicity of ossicular growth marks in a subtropical species. Further, we demonstrate that *C*. *quadricarinatus* ossicles contain easily-extractable chronological information that is suitable for age and growth studies.

Crustacean cuticle comprises four distinct layers: epicuticle, exocuticle, endocuticle and the membranous layer [26–27]. In this study, the ossicular exo- and endocuticle showed isometric growth (Fig 3). It follows that an age-zero ossicle must comprise both layers (e.g. having both exo- and endocuticle), therefore the boundary between them cannot constitute a growth mark. Other crustacean ageing studies have implicitly used this protocol (e.g. [7–8]), but this is the first to provide an evidence-based justification for not counting the cuticular boundary.

Geographical location (i.e. latitude) can affect the interpretability of growth marks in fish otoliths [11]. However, the growth marks in subtropical *C*. *quadricarinatus* ossicles provided repeatable age estimates that were consistently interpreted. Between-reader variation was attributed to the propensity for some ossicles to have a disproportionately well-defined secondary mark series that sometimes produced ambiguous interpretations. Kilada et al. [8] reported that the best structures for ageing *H*. *americanus* were internal structures (i.e. mesocardiac ossicles), but external parts (i.e. eyestalks) were preferred for other temperate-water crustaceans (also see [9–10]). However, we could not find any growth marks in *C*. *quadricarinatus* eyestalks and pterocardiac ossicles were consistently superior to the other ossicles examined. The utility of fish ageing structures varies and is often determined on a species-by-species basis [1]. A similar approach is needed for decapod crustaceans.

Kilada et al. [8] and Weldon et al. [28] reported that only the zygocardiac and mesocardiac tooth plates are moulted. Our observations indicate that the process is the same for *C*. *quadricarinatus*. The tooth plates are subject to heavy wear during intermoult periods and moulting allows for cutting surface renewal and size increase [28]. The absence of moulted ossicles indicates that these structural components are probably retained for life [8, 28]. Like Weldon et al. [28] however, we believe that specifically-tailored investigations into the fate of the gastric mill during ecdysis are needed to promote certainty.

Kilada et al. [8] clearly demonstrated that despite moulting calcein-stained mineral features were retained in the eyestalks and mesocardiac ossicles of *H*. *americanus*. This study has extended this finding to the pterocardiac ossicles of subtropical crustaceans (over an annual cycle). Kilada et al. [8] proposed that the perpetuation of cuticular mineral structure through consecutive moults allows for the accumulation of chronological information in crustaceans. In this study, the sequential addition of urocardiac ridges with increasing *C*. *quadricarinatus* size demonstrated that the new-formed mesocardiac tooth is not simply a larger duplicate and that new information (i.e. urocardiac ridges) is added to the original template. Analogous developmental changes occur in the foregut of *H*. *americanus* larvae [29]. We propose that a similar process could govern the accrual of ossicular growth marks–i.e., previously recorded internal growth rate variations could be duplicated, before being added to over time. Such a memory-like mechanism would explain the existence of chronological information in moulted structures (i.e. eyestalks [8–10]). However, irrespective of whether *C*. *quadricarinatus* ossicles are moulted, their capacity for long-term calcein retention and subsequent deposition of unstained material demonstrates the recording of past events.

The maximum individual *C*. *quadricarinatus* putative age reported here (3+) corroborates that reported from indirect ageing studies [21–22]. Given that juvenile *C*. *quadricarinatus* moult 12 times during their first three months [30], the ossicular growth marks cannot equate to moult-number (also see [8]). For example, the 0+ juveniles in this study would have moulted numerous times, but neither had yet deposited a growth mark. The apparent extension of a near-annual cycle beyond the calcein stain (Fig 7) and the observed maximum number of growth marks both strongly support the annual-deposition hypothesis.

The 1–3+ *C*. *quadricarinatus* sacrificed in autumn had sufficient material deposited beyond the outermost mark to suggest that deposition of a new mark was imminent. The position of the calcein stain (Fig 7) just before a growth mark further supports this observation. In all crustaceans, growth is positively correlated with temperature [31]. It follows that the observed minimum in *C*. *quadricarinatus* growth during winter would coincide with reduced ossicular deposition. Given the above, it appears likely that ossicular growth marks are deposited during winter, but formal studies investigating the timing of growth mark formation are needed.

Similar to other crustaceans (e.g. eastern rock lobster, *Sagmariasus verreauxi* [32]), *C*. *quadricarinatus* size-at-age was asymptotic and could be described using the von Bertalanffy equation. The von Bertalanffy growth parameters presented here for *C*. *quadricarinatus* (from an aquaculture population) are not representative of wild populations. However, the applicability of the von Bertalanffy model to *C*. *quadricarinatus* demonstrates that inexpensive and relatively rapid (e.g. compared with multi-year tagging programs) growth descriptions are possible. The widespread derivation of von Bertalanffy parameters would increase the comparative value of crustacean growth studies at any ecological scale and among captive and wild stocks.

The demonstrated ability to extract age information from subtropical decapods has other global implications. The production of directly-obtained and validated ages would facilitate the development of age-based stock assessments and allow for the quantification of latitudinal-based growth variations in widely-distributed species. In addition, direct ageing studies would facilitate age and growth assessments in fisheries harvesting unusually long-lived species in relatively difficult to access, data-poor or developing fisheries. Similarly, ecological studies on slow-growing crustaceans of conservation concern could be considerably improved, particularly for species where indirect methods probably produce negatively-biased age estimates (e.g. [15–16]). For such species, accurate age information would substantially improve both growth [1] and extinction-threat modelling [33]. Conversely, improved growth and longevity estimates for invasive species (e.g. *C*. *quadricarinatus*) would contribute towards meeting global conservation challenges.

On the balance of evidence, it appears that *C*. *quadricarinatus* growth marks are deposited annually. However, further research utilising longer grow-out periods (e.g. 2 years), larger sample sizes and multiple approaches are required for a definitive validation (see [1]). Assessment of alternative calcein concentrations and exposure durations or less-expensive chemical tags might improve the existing techniques. Increased understanding of the structural nature of ossicular growth marks and the mechanism governing their deposition is needed to promote certainty and facilitate the widespread uptake of direct crustacean ageing methods.
